# Impact of Prosthesis-Patient Mismatch on Hemodynamics During Exercise in Patients With Aortic Stenosis After Transcatheter Aortic Valve Implantation With a Balloon-Expandable Valve

**DOI:** 10.3389/fcvm.2021.799285

**Published:** 2022-01-31

**Authors:** Haruka Kameshima, Masaki Izumo, Tomomi Suzuki, Hiroshi Ohara, Yukio Sato, Mika Watanabe, Shingo Kuwata, Kazuaki Okuyama, Ryo Kamijima, Manabu Takai, Seisyou Kou, Yasuhiro Tanabe, Tomoo Harada, Yoshihiro J. Akashi

**Affiliations:** ^1^Division of Cardiology, Department of Internal Medicine, St. Marianna University School of Medicine, Kawasaki, Japan; ^2^Division of Cardiovascular Medicine, Department of Internal Medicine, Toho University Faculty of Medicine, Tokyo, Japan

**Keywords:** prosthesis-patient mismatch (PPM), aortic stenosis (AS), transcatheter aortic valve implantation (TAVI), exercise induced pulmonary hypertension, exercise stress echocardiography

## Abstract

**Background:**

There is no evidence of hemodynamic performance during exercise in patients with aortic stenosis (AS) after transcatheter aortic valve implantation (TAVI). This study aimed to investigate the changes in kinematic hemodynamics during exercise and determine the impact of prosthesis-patient mismatch (PPM) on the hemodynamics of transcatheter heart valves using exercise stress echocardiography (ESE) in AS patients after TAVI.

**Methods and Results:**

This study enrolled 77 consecutive patients (mean age 82 ± 5 years, 50.6% male) who underwent ESE 3–6 months after TAVI with a balloon-expandable valve. The effective orifice area index at rest was significantly correlated with the mean pressure gradient (PG) during exercise (*p* <0.001). The patients were divided into two groups according to the presence of PPM (PPM and non-PPM groups). During exercise, the patients with PPM had a higher left ventricular ejection fraction (74.6 ± 6.1% vs. 69.7 ± 9.6%, *p* = 0.048), a lower stroke volume index (47.2 ± 14.0 ml/m^2^ vs. 55.6 ± 14.5 ml/m^2^, *p* = 0.037), a significantly higher mean transvalvular PG (21.9 ± 9.1 mmHg vs. 12.2 ± 4.9 mmHg, *p* = 0.01) and an increased mean PG from rest to exercise (5.7 ± 3.5 mmHg vs. 2.3 ± 2.8 mmHg, *p* <0.001) compared with patients without PPM. Patients with PPM had a higher pulmonary artery systolic pressure (SPAP) during exercise (57.3 ± 13.8 mmHg vs. 49.7 ± 10.9 mmHg, *p* = 0.021) and a higher incidence of exercise-induced pulmonary hypertension (43.8 vs. 15.0%, *p* = 0.037) than patients without PPM. PPM was strongly associated with exercise-induced pulmonary hypertension (hazard ratio: 3.570, *p* = 0.013).

**Conclusions:**

AS patients with PPM after TAVI showed a disproportionate increase in the transvalvular PG and SPAP during exercise, and PPM was associated with exercise-induced pulmonary hypertension.

## Introduction

Aortic stenosis (AS) has become a common public health problem in the aging society. Transcatheter aortic valve implantation (TAVI) has changed the paradigm of care for AS patients and is currently being assessed for use in patients with a low surgical risk (1). As the indications for TAVI expand, the age of patients eligible for this type of treatment is decreasing and their level of activity in daily life is increasing accordingly. As a result, transcatheter heart valves (THVs) should have longer durability and better hemodynamic performance.

Prosthesis-patient mismatch (PPM) was first defined in 1978 to describe the mismatch between the hemodynamics of a valve prosthesis and the patient requirements for cardiac output (CO) (2). PPM occurs when the effective orifice area (EOA) of the prosthetic valve is very small in relation to the body surface area (BSA) of the patient, thus resulting in high residual postoperative pressure gradients (PGs) across the prosthesis. This problem is associated with postoperative prognosis (3–6) and prosthetic valve durability (7), and more recently, severe PPM has also been reported to be associated with prognosis after TAVI (6, 8, 9). However, to the best of our knowledge, there is no evidence of changes in hemodynamic performance during exercise in AS patients after TAVI. Therefore, the purpose of this study was to investigate the kinematic hemodynamics during exercise and determine the impact of PPM on the hemodynamics of THVs using exercise stress echocardiography (ESE) in AS patients after TAVI.

## Materials and Methods

### Study Population and Study Design

**Figure 1:**
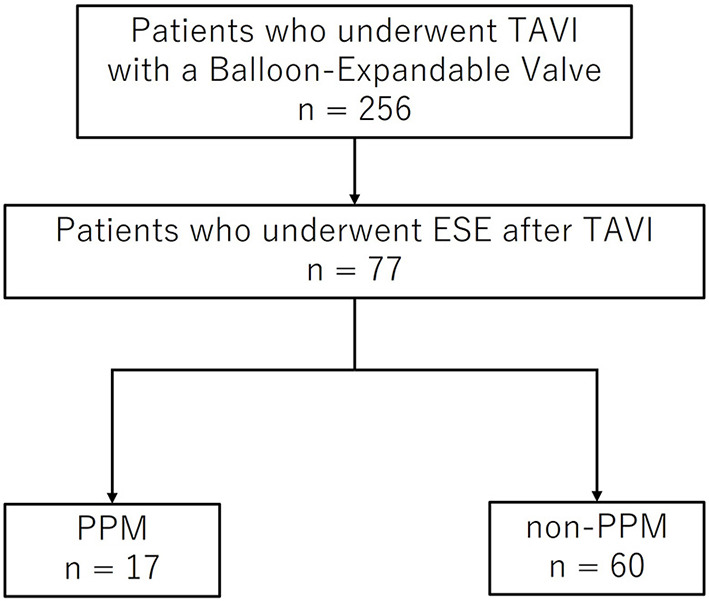
Flow diagram of patient recruitment. TAVI, transcatheter aortic valve implantation; ESE, exercise stress echocardiography; PPM, prosthesis-patient mismatch.

This study retrospectively reviewed 256 consecutive patients who underwent TAVI with a balloon-expandable valve between January 2016 and December 2018 at the St. Marianna University School of Medicine Hospital. The Balloon-expandable valve devices were Sapien XT and Sapien 3 (Edwards Lifesciences, Irvine, CA, USA). Among these, 77 patients who underwent ESE 3–6 months after TAVI were enrolled in our study. Figure 1 is a flow diagram of this study. All TAVI procedures were performed under general anesthesia. The study was performed in accordance with the Declaration of Helsinki and was approved by the Institutional Review Board of the St. Marianna University School of Medicine, Japan (No. 1288). Written informed consent was obtained from all the patients.

### Exercise Stress Protocol

Exercise was performed using the symptom-limited bicycle exercise test in the semi-supine position on a dedicated tilting exercise table at an initial workload of 10 Watt for 3 min, followed by a 10 Watt increase in workload every 3 min. Two-dimensional imaging and Doppler echocardiography data were obtained throughout the exercise test. The endpoints for terminating exercise were as follows: target heart rate reached, symptoms developed; blood pressure of <80 or >220 mmHg; ischemic electrocardiogram changes; ventricular arrhythmia; and rapid atrial tachycardia.

### Transthoracic Echocardiography

Transthoracic echocardiography (Vivid E9; GE Vingmed Milwaukee, WI, USA) was performed at rest and during exercise. All images were digitally stored for offline analysis (EchoPAC, version 12; GE Vingmed Milwaukee, WI, USA), and they included standard two-dimensional, color, pulsed, and continuous-wave Doppler acquisitions according to the current American Society of Echocardiography guidelines (10). Left ventricular (LV) end-diastolic and end-systolic volumes and LV ejection fraction (LVEF) were measured from the standard apical views, according to Simpson's disk summation method. Stroke volume (SV) was measured in the left ventricular outflow tract (LVOT) using the Doppler method, and it was related to the BSA. The CO was obtained by multiplying the SV with heart rate. LV mass was calculated using two-dimensional images and the area-length method (10). To assess LV diastolic function, transmitral early (E-wave) and late (A-wave) velocities were measured using pulsed-wave Doppler imaging at the mitral leaflet tips. The peak early diastolic velocities of the septal mitral annulus (e') were measured using pulsed-wave tissue Doppler imaging from the apical 4-chamber view, and the E/e' ratio was calculated. Suspected pulmonary artery pressure (SPAP) was estimated based on the Doppler spectral signal of the tricuspid regurgitation jet. Pulmonary hypertension (PH) was defined as an SPAP of ≥40 mmHg at rest, and exercise-induced PH was defined as an SPAP of ≥60 mmHg during exercise. PPM was defined as an EOA index (EOAi) of ≤ 0.85 cm^2^/m^2^. The EOA of the THVs was calculated using the continuity equation, according to the current consensus document (11). From a zoomed parasternal long-axis view, the LVOT diameter was measured just below the apical border, i.e., from the outer border to the outer border of the stent or ring. To measure the LVOT flow velocity, the pulsed-wave Doppler was placed immediately below the apical border of the stent, with no valve opening or closing clicks visible. The transprosthetic flow velocity was determined by continuous-wave Doppler imaging with multiwindow interrogation, including the apical and right parasternal windows. The valvulo-arterial impedance (Zva) was calculated using the following formula (12): Zva (mmHg/ml/m^2^) = (mean transvalvular PG [mean PG] + systolic blood pressure)/(SV/BSA). Paravalvular leak was evaluated according to the Valve Academic Research Consortium 3 (13). The patients were divided into two groups according to the presence of PPM (PPM and non-PPM groups).

### Computed Tomography

Preprocedural multidetector computed tomography was performed, and aortic annulus area was measured from 3-dimensional reconstruction recommended by the Society of Cardiovascular Computed Tomography guidelines (14).

### Clinical Outcomes

The primary endpoint of this study was composite outcomes, including all-cause mortality, cardiovascular mortality, cardiovascular event, and heart failure-related hospitalization. The events were determined by reviewing the patients' medical reports or via direct telephonic contact with the patients' families.

### Statistical Analysis

All analyses were performed using SPSS (version 26.0.0, IBM Corporation, Somers, New York). Continuous variables are presented as mean ± standard deviation and were tested for differences using the Student's *t*-test. Categorical variables are presented as frequencies and percentages. The chi-squared and Fisher's exact tests were used to compare the PPM and non-PPM groups. The relationship between the preoperative and postoperative mean PG and the EOAi was evaluated using a simple inverse regression analysis, with r-values (Pearson's correlation coefficient). Survival was estimated using Kaplan-Meier analysis and compared using the two-sided log-rank test. The effects of clinical and echocardiographic parameters were assessed using the Cox proportional hazard model. Statistical significance was set at *p* < 0.05.

## Results

### Clinical and Pre-procedural Echocardiographic Characteristics

Patients with PPM accounted for 17 (22.1%) of the total 77 patients, which included 15 (19.5%) patients with moderate PPM (EOAi of 0.65 cm^2^/m^2^ < and ≤ 0.85 cm^2^/m^2^) and 2 (2.6%) patients with severe PPM (which was defined as an EOAi of ≤ 0.65 cm^2^/m^2^.) The baseline characteristics of the study patients and the procedural characteristics of TAVI are summarized in Table 1 and Supplementary Table 1. The mean age of the study patients was 82 ± 5 years, and 50.6% of the patients were men. The mean Society of Thoracic Surgeons score was 5%, which indicates an intermediate surgical risk. Balloon-expandable THVs of 23 or 26 mm size were used in most patients. Patients with PPM were younger than patients without PPM, and no significant differences were found in sex between the PPM and non-PPM groups. Although the preoperative functional status in terms of the New York Heart Association (NYHA) functional class was similar between the groups, the post-procedure NYHA functional class tended to be higher in patients with PPM than in those without PPM; however, the difference was not statistically significant. The device size used in the PPM group was smaller than that used in the non-PPM group.

**Table 1:** Procedural results and baseline characteristics.

	**All (*n* = 77)**	**PPM (*n* = 17)**	**Non-PPM (*n* = 60)**	***p*-value (PPM vs. non-PPM)**
Age, years	82 ± 5	80 ± 6	83 ± 4	0.003
Men, *n* (%)	39 (50.6)	7 (41.2)	32 (53.3)	0.742
Body surface area, m^2^	1.50 ± 0.17	1.50 ± 0.13	1.50 ± 0.18	0.855
Hypertension, *n* (%)	65 (84.4)	12 (15.6)	53 (88.3)	0.085
Diabetes, *n* (%)	16 (20.8)	3 (17.6)	13 (21.7)	0.507
Hypercholesterolemia, *n* (%)	51 (66.2)	11 (64.7)	40 (66.7)	0.548
Chronic kidney disease, *n* (%)	51 (66.2)	10 (58.8)	41 (68.3)	0.325
Atrial fibrillation, *n* (%)	31 (40.3)	6 (35.3)	25 (41.7)	0.428
Coronary artery disease, *n* (%)	27 (35.1)	3 (17.6)	24 (40.0)	0.075
Myocardial infarction, *n* (%)	6 (7.8)	2 (11.8)	4 (6.7)	0.397
Pre procedure NYHA class				0.554
II, *n* (%)	47 (61.0)	12 (70.6)	35 (58.3)	
III-IV, *n* (%)	28 (36.4)	5 (29.4)	23 (38.3)	
Post procedure NYHA class				0.467
I, *n* (%)	64 (83.1)	13 (76.5)	51 (85.0)	
II, *n* (%)	13 (16.9)	4 (23.5)	9 (15.0)	
III-IV, *n* (%)	0 (0)	0 (0)	0 (0)	
STS score	5.01 ± 2.64	4.45 ± 2.21	5.17 ± 2.74	0.324
Annulus area, mm^2^	425 ± 89	365 ± 64	442 ± 89	0.001
**THV size**				0.001
20 mm, *n* (%)	5 (6.5)	3 (17.6)	2 (3.3)	
23 mm, *n* (%)	36 (46.8)	13 (76.5)	23 (38.3)	
26 mm, *n* (%)	31 (40.3)	1 (5.9)	30 (50.0)	
29 mm, *n* (%)	5 (6.5)	0 (0)	5 (8.3)	
**Approach**				0.276
Trans-femoral, *n* (%)	72 (93.5)	17 (100)	55 (91.7)	

*Data presented as mean ± standard deviation or n (%). PPM, prosthesis-patient mismatch; CABG, coronary artery bypass grafting; NYHA, New York Heart Association; STS, The Society of Thoracic Surgeons; THV, transcatheter heart valve*.

Table 2 presents the preoperative echocardiographic findings. The mean LVEF was not significantly different between patients with PPM and those without PPM. The LV end-diastolic volume and LV mass index were smaller in patients with PPM than in those without PPM. Although the peak velocity was higher in patients with PPM (4.8 ± 1.4 m/s vs. 4.1 ± 1.0 m/s, *p* = 0.030), mean PG and aortic valve area index were not significantly different between the two groups.

**Table 2:** Preoperative echocardiography characteristics.

	**All (*n* = 77)**	**PPM (*n* = 17)**	**Non-PPM (*n* = 60)**	***p*-value (PPM vs. non-PPM)**
LVEDV, ml	85 ± 33	78 ± 21	87 ± 36	0.349
LVESV, ml	32 ± 23	26 ± 11	34 ± 25	0.263
LVEF, %	65.4 ± 10.1	66.9 ± 6.6	64.9 ± 10.9	0.465
SVi, ml/m^2^	42.0 ± 11.7	41.0 ± 8.3	42.3 ± 12.5	0.687
LVMi, g/m^2^	108 ± 30	109 ± 28	108 ± 31	0.883
E/A	0.87 ± 0.90 (*n* = 61)	0.87 ± 0.59 (*n* = 14)	0.87 ± 0.98 (*n* = 47)	0.977
E/e'	16.6 ± 7.2	16.7 ± 7.7	16.6 ± 7.1	0.961
SPAP, mmHg	32.1 ± 9.6	33.0 ± 10.1	31.8 ± 9.5	0.645
Peak velocity, m/s	4.3 ± 1.1	4.8 ± 1.4	4.1 ± 1.0	0.030
Mean PG, mmHg	44.9 ± 23.9	56.1 ± 32.3	41.7 ± 20.1	0.097
AVA, cm^2^	0.64 ± 0.19	0.57 ± 0.16	0.66 ± 0.19	0.087
AVAi, cm^2^/m^2^	0.42 ± 0.12	0.38 ± 0.10	0.44 ± 0.12	0.064

*Data presented as mean ± standard deviation or n (%). LVEF, left ventricular ejection fraction; LVEDV, left ventricular end-diastolic volume; LVESV, left ventricular end-systolic volume; SVi, stroke volume index; CO, cardiac output; LVMi, left ventricular mass index; SPAP, systolic pulmonary artery pressure; Mean PG, mean transvalvular pressure gradient; AVA, aortic valve area; AVAi, aortic valve area index. The other abbreviations are shown in Table 1*.

### Impact of PPM on Hemodynamics at Rest and During Exercise

**Figure 2:**
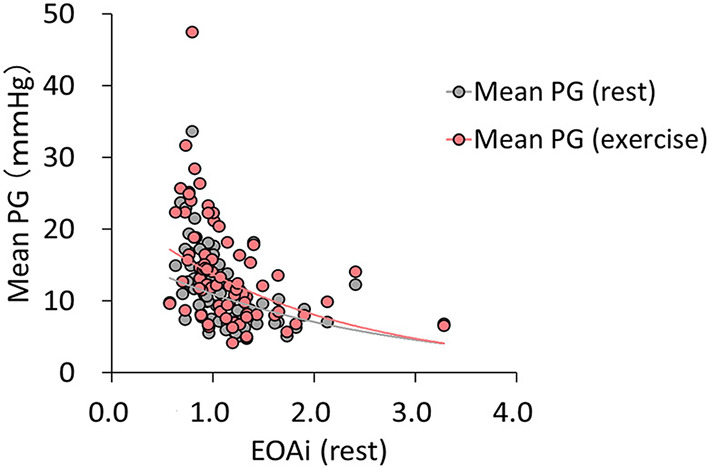
The relationship between the mean PG and EOAi at rest and during exercise. Inverse simple regression analysis of the mean PG and EOAi at rest (*r* = 0.421; *p* < 0.001) and during exercise (*r* = 0.440; *p* < 0.001). Mean PG, mean transvalvular pressure gradient; EOAi, effective orifice area index.

**Figure 3:**
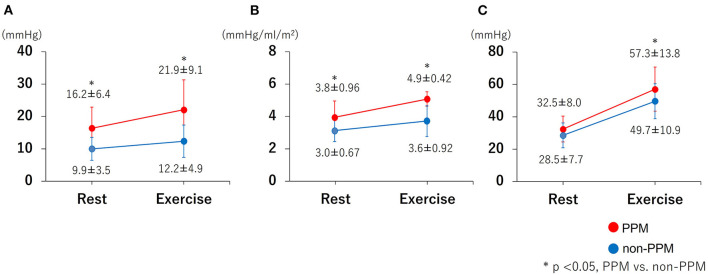
The changes in the mean PG, Zva and SPAP from rest to exercise. **(A)** mean PG increase from rest to exercise; **(B)** Zva increase from rest to exercise; **(C)** SPAP increase from rest to exercise. The increase in the mean PG and Zva from rest to exercise was greater in patients with PPM than in those without PPM. Patients with PPM had a higher SPAP increase than patients without PPM (57.3 ± 13.8 mmHg vs. 49.7 ± 10.9 mmHg). PPM = prosthesis-patient mismatch; Mean PG, mean transvalvular pressure gradient; Zva, valvulo-arterial impedance; SPAP, systolic pulmonary artery pressure. *P*-value, PPM vs. non-PPM.

The hemodynamic characteristics at rest and during exercise are summarized in Tables 3, 4 and in Figures 2, 3. The relationship between the mean PG and EOAi was curvilinear, with a correlation coefficient (*r*) of 0.421 (*p* < 0.001) at rest and 0.440 (*p* < 0.001) during exercise (Figure 2). Exercise capacity was slightly higher in patients without PPM than in those with PPM, although the difference was not statistically significant. At rest, patients with PPM demonstrated a higher peak transvalvular velocity and mean PG than patients without PPM (both *p* ≤ 0.001). Zva and the changes in the mean PG from rest to exercise were significantly greater in patients with PPM than in patients without PPM (Zva: 4.9 ± 1.8 mmHg/ml/m^2^ vs. 3.6 ± 0.92 mmHg/ml/m^2^, Δ mean PG: 5.7 ± 3.5 mmHg vs. 2.3 ± 2.8 mmHg, both *p* ≤ 0.001, Figures 3A,B). Although there were no significant differences in the SPAP and the prevalence of PH at rest between patients with and without PPM, patients with PPM had a higher SPAP and a higher prevalence of exercise-induced PH than patients without PPM (Figure 3C; Table 4).

**Table 3:** Resting echocardiography data.

	**All (*n* = 77)**	**PPM (*n* = 17)**	**Non-PPM (*n* = 60)**	***p*-value (PPM vs. non-PPM)**
Systolic BP, mmHg	139 ± 22	141 ± 25	138 ± 21	0.596
Diastolic BP, mmHg	68 ± 15	72 ± 14	68 ± 16	0.361
Heart rate, beats/min	67 ± 11	66 ± 10	68 ± 10	0.620
LVMi, g/m^2^	84 ± 24	84 ± 21	83 ± 24	0.889
LVEDV, ml	85 ± 29	77 ± 20	88 ± 31	0.211
LVESV, ml	31 ± 19	25 ± 8	33 ± 21	0.099
LVEF, %	65.4 ± 10.1	67.4 ± 8.1	63.6 ± 8.8	0.110
SVi, ml/m^2^	49.2 ± 12.3	43.2 ± 11.9	50.9 ± 12.0	0.023
CO, ml/min	4.9 ± 1.2	4.2 ± 1.0	5.0 ± 1.1	0.008
E/A	0.72 ± 0.18 (*n* = 58)	0.79 ± 0.17 (*n* = 14)	0.79 ± 0.18 (*n* = 44)	0.105
E/e'	21.8 ± 9.9	23.0 ± 9.3	21.5 ± 10.1	0.575
SPAP, mmHg	29.4 ± 7.9	32.5 ± 8.0	28.0 ± 7.7	0.070
TAPSE/SPAP	0.64 ± 0.26	0.57 ± 0.24	0.66 ± 0.26	0.228
Pulmonary hypertension, *n* (%)	7 (9.1)	3 (17.6)	4 (6.7)	0.182
Peak velocity, m/s	2.3 ± 0.46	2.7 ± 0.52	2.2 ± 0.37	<0.001
Mean PG, mmHg	11.3 ± 5.0	16.2 ± 6.4	9.9 ± 3.5	0.001
Zva, mmHg/ml/m^2^	3.2 ± 0.81	3.8 ± 0.96	3.0 ± 0.67	<0.001
PVL	1.3 ± 0.98	0.91 ± 1.0	1.4 ± 0.95	0.066
EOAi, cm^2^/m^2^	1.08 ± 0.31	0.74 ± 0.07	1.17 ± 0.28	<0.001

*Data presented as mean ± standard deviation or n (%). BP, blood pressure; TAPSE, tricuspid annular plane systolic excursion; Mean PG, mean transvalvular pressure gradient; Zva, valvulo-arterial impedance; PVL, para-valvular leak; EOA, effective orifice area; EOAi, effective orifice area index. The other abbreviations are shown in Tables 1, 2*.

**Table 4:** Exercise stress echocardiography data.

	**All (*n* = 77)**	**PPM (*n* = 17)**	**Non-PPM (*n* = 60)**	***p*-value (PPM vs. non-PPM)**
Exercise duration, min	10.3 ± 3.6	9.7 ± 2.4	10.5 ± 3.9	0.274
Peak watt, Watt	32 ± 14	32 ± 10	32 ± 14	0.876
Systolic BP, mmHg	180 ± 30	187 ± 21	178 ± 32	0.153
Diastolic BP, mmHg	76 ± 16	84 ± 15	74 ± 16	0.015
Heart rate, beats/min	104 ± 18	107 ± 21	103 ± 17	0.354
LVEDV, ml	93 ± 31	82 ± 23	95 ± 32	0.128
LVESV, ml	29 ± 20	21 ± 10	31 ± 22	0.094
LVEF, %	70.8 ± 9.1	74.6 ± 6.1	69.7 ± 9.6	0.048
SVi, ml/m^2^	53.7 ± 14.7	47.2 ± 14.0	55.6 ± 14.5	0.037
CO, ml/min	8.2 ± 2.5	7.5 ± 2.5	8.4 ± 2.5	0.216
E/A	1.00 ± 0.24 (*n* = 49)	0.99 ± 0.22 (*n* = 13)	1.00 ± 0.26 (*n* = 36)	0.853
E/e'	21.9 ± 7.9	23.2 ± 9.2	21.5 ± 7.6	0.465
SPAP, mmHg	51.5 ± 12.0	57.3 ± 13.8	49.7 ± 10.9	0.021
TAPSE/SPAP	0.48 ± 0.15	0.44 ± 0.10	0.49 ± 0.16	0.230
Exercise-induced pulmonary hypertension, *n* (%)	16 (20.8)	7 (43.8)	9 (15.0)	0.037
Peak velocity, m/s	2.5 ± 0.58	3.0 ± 0.7	2.4 ± 0.5	<0.001
Mean PG, mmHg	14.3 ± 7.3	21.9 ± 9.1	12.2 ± 4.9	0.001
Δ Mean PG, mmHg	3.1 ± 3.2	5.7 ± 3.5	2.3 ± 2.8	<0.001
Zva, mmHg/ml/m^2^	3.9 ± 1.2	4.9 ± 1.8	3.6 ± 0.92	0.010
Δ Zva, mmHg/ml/m^2^	0.69 ± 1.1	1.1 ± 1.7	0.57 ± 0.89	0.243

*Data presented as mean ± standard deviation or n (%). Δ Zva, meant the change in Zva from rest to exercise. The other abbreviations are shown in Tables 1, 2*.

The results of univariate Cox proportional hazard analyses for the prediction of exercise-induced PH are presented in Table 5. Age and preoperative aortic valve area index (AVAi) were associated with exercise-induced PH. PPM was strongly associated with exercise-induced PH. Although there was no significant association between exercise-induced PH and mean PG, Zva, E/e' and blood pressure were associated with exercise-induced PH both at rest and during exercise. The echocardiographic parameters of right-side heart function, such as tricuspid annular plane systolic excursion or tricuspid annular plane systolic excursion/SPAP, were similar in the PPM and non-PPM groups.

**Table 5:** Univariate models of Cox regression analysis for exercise induced pulmonary hypertension.

	**Univariate**		
	**HR**	**95% CI**	***p*-value**
**Patient characteristics**			
Female sex	1.020	0.375–2.769	0.970
Age	0.902	0.824–0.987	0.024
Atrial fibrillation	0.816	0.281–2.367	0.708
**Preoperative echocardiography**			
SVi	0.986	0.943–1.031	0.529
SPAP	1.019	0.976–1.063	0.401
E/A	1.120	0.575–2.184	0.738
E/e'	1.064	0.993–1.140	0.078
AVAi	0.006	0.000–0.909	0.046
Mean PG	1.010	0.995–1.026	0.199
**Exercise echocardiography**			
Rest systolic BP	1.038	1.013–1.063	0.003
Rest diastolic BP	1.031	1.008–1.054	0.007
Rest SVi	1.002	0.957–1.048	0.942
Rest CO	0.923	0.554–1.537	0.758
Rest SPAP	1.063	1.019–1.110	0.005
Rest E/A	35.132	3.062–403.1	0.004
Rest E/e'	1.082	1.019-1.148	0.009
PPM	3.570	1.304–9.778	0.013
Rest Mean PG	1.058	0.997–1.123	0.064
Rest Zva	2.025	1.108–3.701	0.022
Peak systolic BP	1.040	1.015–1.065	0.001
Peak diastolic BP	1.091	1.044–1.140	<0.001
SVi during exercise	0.985	0.953–1.019	0.386
CO during exercise	0.994	0.834–1.183	0.943
E/A during exercise	1.112	0.086–14.41	0.935
E/e' during exercise	1.087	1.019–1.159	0.011
Mean PG during exercise	1.039	0.998–1.082	0.066
Zva during exercise	1.728	1.179–2.534	0.005

*HR, hazard ratio; CI, confidence interval. The other abbreviations are shown in Tables 1–3*.

**Figure 4:**
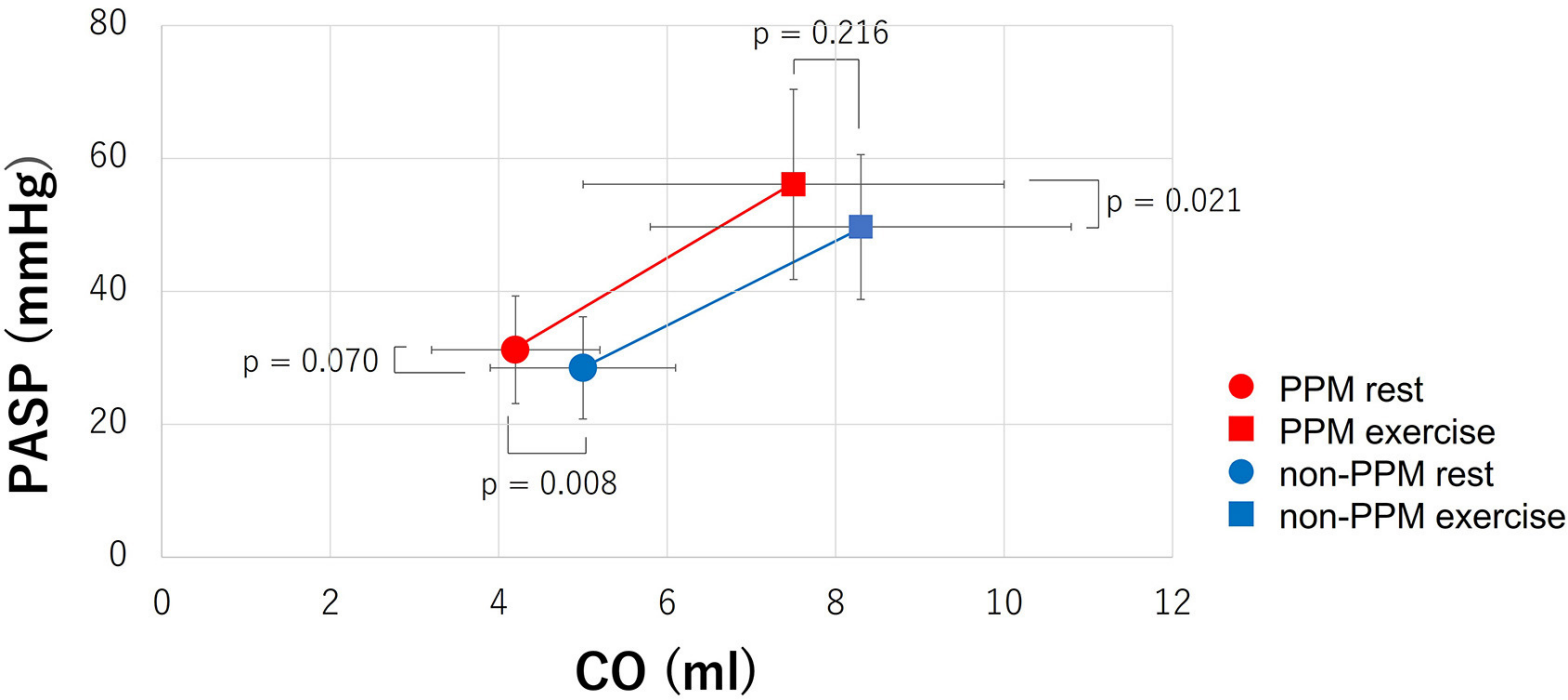
The relationship between the SPAP and CO at rest and during exercise. The CO tended to be lower and the SPAP was higher in patients with PPM than in those without PPM both at rest and during exercise. The slopes showed that ΔSPAP/ΔCO was higher in patients with PPM than in those without PPM, but the difference was not statistically significant (23.9 ± 45.9 vs. 8.9 ± 14.4, *p* = 0.202).SPAP = systolic pulmonary artery pressure; CO, cardiac output; PPM, prosthesis-patient mismatch; ΔSPAP/ΔCO, the ratio of the change in SPAP and the change in CO.

The CO at rest was lower in patients with PPM than in patients without PPM, although the difference was not statistically significant during exercise. Figure 4 depicts the relationship between the SPAP and CO at rest and during exercise. According to the diagram, the CO tended to be lower and the SPAP was higher in patients with PPM than in those without PPM both at rest and during exercise. The slopes showed that the ratio of the change in the SPAP and the change in the CO (ΔSPAP/ΔCO) was higher in patients with PPM than in those without PPM, but the difference was not statistically significant (23.9 ± 45.9 vs. 8.9 ± 14.4, *p* = 0.202).

### Prognostic Impact of PPM

**Figure 5:**
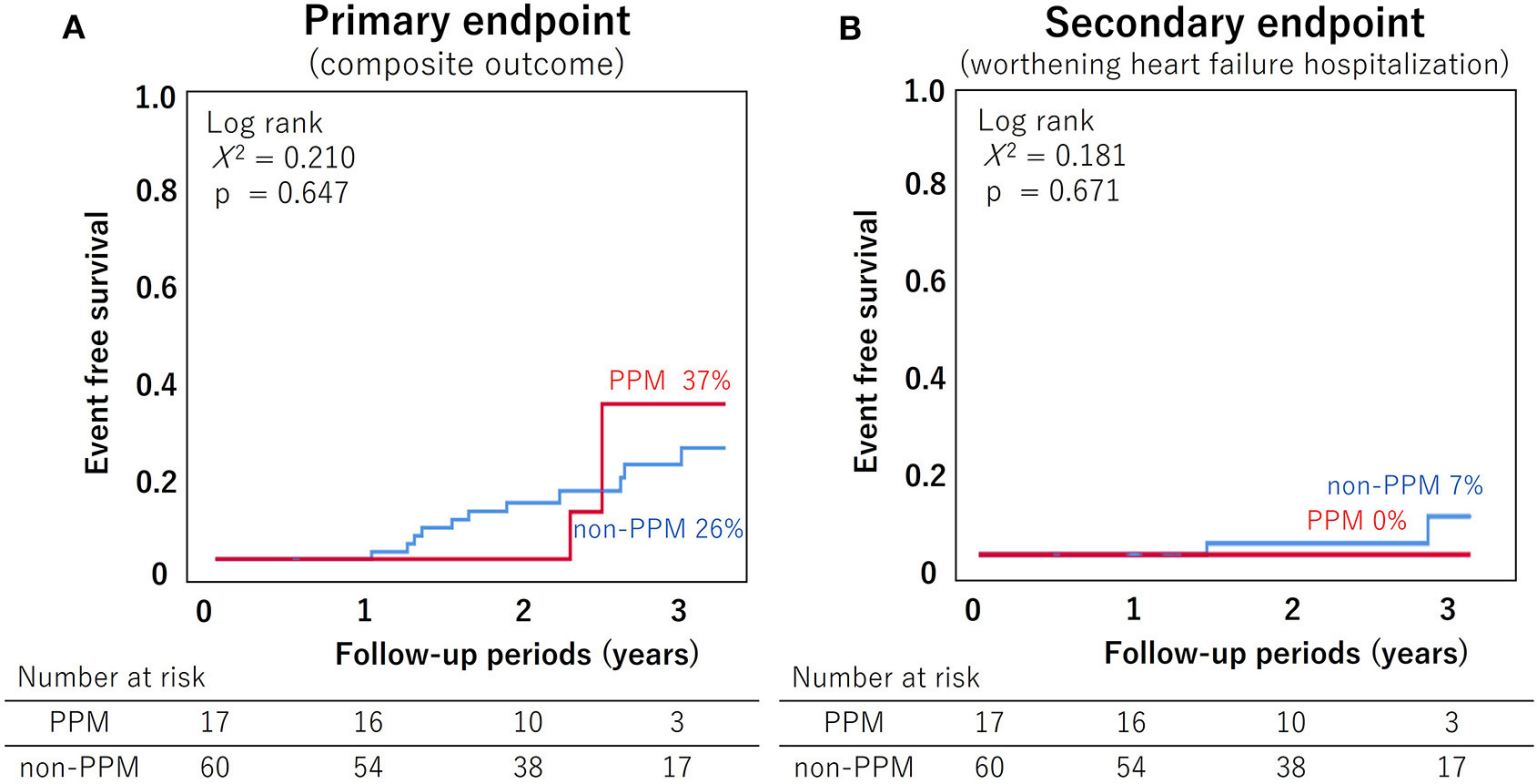
The Kaplan-Meier survival curves for all-cause mortality and heart failure-related hospitalization. **(A)** Primary endpoint (composite outcomes including all-cause mortality, cardiovascular mortality, cardiovascular event, and heart failure-related hospitalization); **(B)** Secondary endpoint (heart failure-related hospitalization). There were no differences in the primary and secondary endpoints between the PPM and non-PPM groups. PPM, prosthesis-patient mismatch.

During the 28 ± 10 months follow-up period, 19 patients reported the primary composite endpoint and five patients had the secondary endpoint of heart failure-related hospitalization. Kaplan-Meier survival curves (Figure 5) did not show any significant differences in both primary and secondary endpoints between patients with and without PPM (primary endpoint: log-rank χ^2^ = 0.210, *p* = 0.647; secondary endpoints: log-rank χ^2^ = 0.181, *p* = 0.671).

## Discussion

The major findings of this study are as follows: (1) The EOAi at rest correlated well with the mean PG at rest and during exercise; (2) patients with PPM showed a disproportionate increase in the transvalvular PG, LV afterload, and SPAP during exercise compared with patients without PPM; (3) the prevalence of exercise-induced PH after TAVI was higher in the PPM group than in the non-PPM group, and the improvement of symptoms tended to be poor in the PPM group; and (4) PPM was not associated with all-cause mortality or heart failure-related hospitalization during 2 years in this cohort.

### Hemodynamic Changes During Exercise

Some previously published reports (15–17) have addressed the hemodynamic characteristics of various surgical aortic prosthetic valves during exercise. To the best of our knowledge, this is the first study to assess the hemodynamic characteristics of THVs during exercise using ESE. Pibarot (15, 16) reported that PPM is associated with a marked increase in the mean PG and the prevalence of PH, whereas a normal functional prosthetic valve shows a minimal increase in the mean PG. These findings regarding surgical prosthesis were consistent with the major findings of our study that included AS patients after TAVI. O'Sullivan et al. (18) demonstrated that postcapillary PH (left-sided PH) is the most common form of PH among patients with severe symptomatic AS undergoing TAVI. In postcapillary PH, the increased SPAP is mainly thought to be due to the passive backward transmission of an increased LV filling pressure (19, 20). Although the SPAP usually correlates with the CO, our study also found that patients with PPM had a higher SPAP during exercise despite a lower CO than patients without PPM. These findings suggest that the LV afterload during exercise was higher in the PPM group than in the non-PPM group and that the impaired LV diastolic function, increased LV filling and left atrial pressure are causes of disproportionate increase in pulmonary artery pressure. However, no statistically significant difference was noted in the diastolic function parameters, including the E/A ratio in our study. Despite of the fact that the mitral variables the mitral variables of the E/A ratio are the main parameters for evaluating diastolic function, many studies reported there is a weak correlation between the echocardiographic indices of the LV diastolic function and the LV filling pressure in patients with LV hypertrophy (21–23). In addition, the presence of mitral annular calcification reportedly underestimates the e' value (24, 25) in this study, mitral annular calcification was observed in ~30% of the patients, which is considered to be one of the reasons for the lack of difference in the E/e' ratio.

### Clinical Impact of PPM

Many studies have documented the negative clinical impact of PPM after surgical aortic valve replacement (SAVR) and TAVI, focusing on clinical outcomes such as survival (3–6, 8, 9), heart failure worsening (8), LV mass regression (5, 6), and valve durability (7). Bleiziffer S (17) reported that the presence of PPM significantly influences the percentage of predicted exercise capacity and impairs the quality of life in patients. Although no statistically significant difference was found in our study in terms of adverse events, the post-procedure NYHA functional class was higher in patients with PPM than in patients without PPM. The transvalvular PG and SPAP were higher in patients with PPM than in patients without PPM, and the prevalence of exercise-induced PH was higher in patients with PPM (Figure 3), which resulted in a higher NYHA functional class. We speculated that an increased hemodynamic burden due to higher gradients in patients with PPM could explain this phenomenon. Another possible explanation is that PPM may limit the increase in the CO (Figure 4). This may, in turn, limit the capacity of cardiac function to match the increasing metabolic demand during exercise.

In our study, there were no differences in the hard endpoints between the PPM and non-PPM groups (Figure 5). The reasons for this possibly include the short follow-up period, elderly patients, and inclusion of exercisable patients only. It is important to estimate the postoperative EOA and predict PPM from preoperative cardiac imaging. In this study, prosthetic valve EOA tended to increase with preoperative annulus area, and each value was similar to the previously reported valve area in the CoreLab analysis (26) (Supplementary Table 1). It is desirable to predict prosthetic valve size and determine the predicted EOA and PPM from preoperative imaging data and select treatment strategy accordingly.

A previous study with a similar follow-up in AS patients after TAVI reported that only severe PPM was associated with prognosis (6, 8). Moreover, Schofer et al. (9) reported that in patients with low LVEF (<40%), severe PPM was associated with increased risk of mortality. In our study, severe PPM was noted in only two patients, and no patient had low LVEF or severe PPM.

## Limitations

This study was performed with a relatively small number of patients. Nonetheless, our findings are new, and important implications were made regarding the behavior of the transcatheter aortic valve during exercise. In this study, only balloon-expandable THV was examined, and the study of hemodynamics during exercise in patients with Self-expandable THVs and comparison with prosthetic valve types is warranted. It is unknown whether ESE performed at 3–6 months after TAVI is valid in terms of the time period for evaluating the impact of PPM on hemodynamics. Most patients in our study were elderly; therefore, it was difficult to perform ESE in such patients after long periods. Some patients, including young and low-risk patients, in previous studies (17) underwent ESE from 5 to 12 months after SAVR. Further studies are necessary to investigate PPM in post-TAVI patients after longer periods.

## Conclusion

AS patients with PPM after TAVI showed a disproportionate increase in the transvalvular gradient and SPAP during exercise. In addition, PPM was associated with exercise-induced PH.

## Data Availability Statement

The original contributions presented in the study are included in the article/Supplementary Material, further inquiries can be directed to the corresponding author/s.

## Ethics Statement

The studies involving human participants were reviewed and approved by Institutional Review Board of the St. Marianna University School of Medicine. The patients/participants provided their written informed consent to participate in this study.

## Author Contributions

MI contributed to conception and design of the study. HK performed the statistical analysis. HK and MI wrote the manuscript. YA helped revise the manuscript. All authors organized the database and read and approved the submitted version.

## Conflict of Interest

MI is a screening proctor for Edwards Lifesciences. The remaining authors declare that the research was conducted in the absence of any commercial or financial relationships that could be construed as a potential conflict of interest.

## Publisher's Note

All claims expressed in this article are solely those of the authors and do not necessarily represent those of their affiliated organizations, or those of the publisher, the editors and the reviewers. Any product that may be evaluated in this article, or claim that may be made by its manufacturer, is not guaranteed or endorsed by the publisher.
