# Supplementary material for: Impact of Prosthesis-Patient Mismatch on Hemodynamics During Exercise in Patients With Aortic Stenosis After Transcatheter Aortic Valve Implantation With a Balloon-Expandable Valve
Source: Front Cardiovasc Med. 2022 Jan 31;8:799285. doi: 10.3389/fcvm.2021.799285 (PMC8841769; doi:10.3389/fcvm.2021.799285)
Supplement: Supplementary file 1 [file Table_1.docx]

**Supplemental Table 1**

| Annulus area, mm^2^ | 248-384 | 385-439 | 440-488 | 489-537 | 538-678 | p value |
| --- | --- | --- | --- | --- | --- | --- |
| n | 24 | 18 | 6 | 5 | 6 |  |
| EOA, cm^2^ | 1.30 ± 0.31 | 1.63 ± 0.46 | 1.87 ± 0.31 | 1.57 ± 0.39 | 1.90 ± 0.42 | 0.001 |
| EOAi, cm^2^/m^2^ | 0.93 ± 0.27 | 1.12 ± 0.38 | 1.23 ± 0.33 | 0.95 ± 0.22 | 1.13 ± 0.27 | 0.131 |
| PPM, n (%) | 12 (50.0) | 4 (22.2) | 0 (0) | 1 (20.0) | 0 (0) | - |

Data presented as mean ± standard deviation or n (%). EOA = effective orifice area; EOAi = effective orifice area index; PPM = prosthesis-patient mismatch.
